# The ISCB: Growing and Evolving in Step with Science

**DOI:** 10.1371/journal.pcbi.0010051

**Published:** 2005-10-28

**Authors:** Michael Gribskov

In 1993, the Intelligent Systems for Molecular Biology conference was successfully launched as an annual meeting for scientific exchange within the nascent interdisciplinary science of computational biology. Demand grew stronger each year for an umbrella organization that extended its reach beyond a once-a-year conference. The International Society for Computational Biology (ISCB) was formed in 1997 to provide computational biologists and bioinformaticians worldwide with a community of peers with whom they could interact year-round in their mutual quest to advance the understanding of living systems through computation. Over the past 13 years, the ISCB has grown and evolved along with the fields of computational biology and bioinformatics. As the society's president, I am proud to report that our membership now comprises nearly 2,000 members in over 50 countries.

Each year, *PLoS Computational Biology,* as the official journal of the ISCB, will publish the bylaws of the society. These are the legally registered rules by which the elected and appointed leaders of the society, along with the membership at large, must abide. It is my hope that ISCB members will take the time to read these rules, and to understand, thereby, the legal framework in which ISCB operates. Clearly, as ISCB grows, these rules will be adapted to changing circumstances—knowing the current rules is a first step to making any improvements to them. We also believe that our bylaws may be useful to other groups trying to form similar organizations.

## International Society for Computational Biology Bylaws

### Article I: Office

#### Section 1: Principal office.

The principal office of the Corporation shall be at San Diego Supercomputer Center, UCSD, 9500 Gilman Drive, La Jolla, CA 92093-0505

#### Section 2: Other offices.

The Corporation may also have an office or offices in such other place or places as the business of the Corporation may require and the Board of Directors may from time to time appoint.

### Article II: Members

#### Section 1:Annual meeting.

The annual meeting of the members of the Corporation shall be held on a day duly designated by the Board of Directors either within or without the United States if not a legal holiday, and if a legal holiday then the next succeeding day not a legal holiday, for the transaction of such corporate business as may come before the meeting.

#### Section 2: Special meetings.

Special meetings of the members may be called at any time for any purpose or purposes by the Chairman of the Board, the President, a Vice President, or a majority of the Board of Directors, and shall be called forthwith by the Chairman of the Board, the President, a Vice President, the Secretary or any Director of the Corporation upon the request in writing of a majority of all the members entitled to vote on the business to be transacted at such meeting. Such request shall state the purpose or purposes of the meeting. Business transacted at all special meetings of members shall be confined to the purpose or purposes stated in the notice of the meeting.

#### Section 3: Place of holding meetings.

All meetings of members shall be held within or without the United States at a place designated by the Board of Directors. The members may hold their meetings in person, by conference telephone, by E-mail over the Internet or other similar electronic media, or by any combination of the foregoing.

#### Section 4: Notice of meetings.

Written notice of each meeting of the members shall be mailed, postage prepaid by the Secretary or person appointed by the President or sent by E-mail over the Internet or similar electronic media by the Secretary or person appointed by the President, to each member of record entitled to vote thereat at his or her post office address or E-mail address, as it appears upon the books of the Corporation, at least ten (10) days before the meeting. Each such notice shall state the place, E-mail information if the meeting will be held partly or completely by electronic means over the Internet or other electronic media, day, and hour at which the meeting is to be held and, in the case of any special meeting, shall state briefly the purpose or purposes thereof.

#### Section 5: Quorum.

The presence in person, by E-mail over the Internet or similar electronic media, or by proxy (each of which shall constitute “Attendance” for all purposes of these bylaws), of one third of the members of the Corporation shall constitute a quorum at all meetings of the members except as otherwise provided by law, by the Certificate of Incorporation or by these bylaws If less than a quorum shall be in Attendance at the time for which the meeting shall have been called, the meeting may be adjourned from time to time by a majority vote of the members in Attendance, without any notice other than by announcement at the meeting, until a quorum shall be in Attendance. At any adjourned meeting at which a quorum shall be in Attendance, any business may be transacted which might have been transacted if the meeting had been held as originally called.

#### Section 6: Conduct of meetings.

Meetings of members shall be presided over by the President of the Corporation or, if he is not in Attendance, by a Vice President, or, if none of said officers is in Attendance, by a chairman to be elected at the meeting. The Secretary of the Corporation, or if he is not in Attendance, any Assistant Secretary shall act as secretary of such meetings; in the absence of the Secretary and any Assistant Secretary, the presiding officer may appoint a person to act as Secretary of the meeting.

#### Section 7: Voting.

At all meetings of members every member entitled to vote thereat shall have one (1) vote. Such vote may be made either in person, by conference call, by E-mail over the Internet or similar electronic media, or by proxy appointed by an instrument in writing subscribed by such member or his or her duly authorized attorney, bearing a date not more than three (3) months prior to said meeting, unless said instrument provides for a longer period. Such proxy shall be dated, but need not be sealed, witnessed or acknowledged. All elections shall be had and all questions shall be decided by a majority of the votes cast at a duly constituted meeting, except as otherwise provided by law, in the Certificate of Incorporation or by these bylaws.

If the chairman of the meeting shall so determine, a vote by ballot may be taken upon any election or matter, and the vote shall be so taken upon the request of ten percent (10%) or more of all of the members entitled to vote on such election or matter. In either of such events, the proxies and ballots shall be received and be taken in charge and all questions touching the qualification of voters and the validity of proxies and the acceptance or rejection of votes, shall be decided by members appointed by the chairman of said meeting.

#### Section 8: Identity of members.

The members of the Corporation shall be composed of those individuals who have filled out a registration form and paid their dues. Individuals shall retain their status as members so long as they pay any and all annual dues imposed by the Corporation upon its members.

#### Section 9: Members' voting rights.

The members of the Corporation shall nominate from the membership, no later than their annual meeting in the manner set forth in the Procedure for Nomination, up to four (4) Directors who shall also be concurrently nominated to hold one of the Nominated Offices as further defined in Article IV, Section 1 below. Said nominations shall be considered strong recommendations from the members to the Board of Directors to elect these individuals as officers and Directors of the Corporation (the “Director/Officers”). Upon their election by the Directors as set forth in Article III, Section 4 below, these four Director/Officers shall serve as officers of the Corporation for one year in the case of President-elect, and two years for all other officers including President. The four Director/Officers shall also concurrently serve as Directors of the Corporation during their full term as officers plus one year following completion of their term or terms of office, unless removed by the Directors pursuant to the provisions of Article III, Section 4(iv) below. The membership shall follow such rules pertaining to the nomination of officers as are promulgated by the Board of Directors in the Procedure For Nomination adopted as of March 2003 as attached to these bylaws as Exhibit A, or as later amended.

#### Section 10: Directors as members.

The Board of Directors of the Corporation shall be members. A Director who fails to keep his or her membership current during the course of his or her term will forfeit voting rights until current year registration is rectified, or resign from the Board of Directors if not registered by April 1 of the membership year.

### Article III: Board of Directors

#### Section 1: General powers.

The property and business of the Corporation shall be managed under the direction of the Board of Directors of the Corporation.

#### Section 2: Number.

The number of Directors, including Director/Officers, shall be twenty eight (28) or such other number, but not less than three (3) nor more than thirty (30), as may be designated from time to time by resolution of a majority of the entire Board of Directors.

#### Section 3: Term of office.

The term of office of each Director, other than a Director/Officer, shall be for three (3) years; the term of office of each Director/Officer shall be for the duration of the term or terms of office of the officer plus one additional year after the final term as officer.

#### Section 4: Filling of vacancies.

- In the case of any vacancy in the Board of Directors, including Director/Officers, the remaining Directors may elect a successor at a regular or special meeting of Directors, by affirmative vote of the majority thereof.
- In the case of any vacancy in the Director/Officers, the Directors may, but are not required to, ask the members to make nominations under the Procedure for Nomination.
- If the number of Directors is increased as provided in these bylaws, the additional Directors so provided for shall be elected by an affirmative vote of a majority of the entire Board of Directors already in office to hold office for a three (3) year term.
- Notwithstanding the foregoing provisions, any Director, including a Director/Officer, may be removed from office as a Director with or without cause by the affirmative vote of a majority of the Directors entitled to vote at any regular or special meeting of Directors called for that purpose. A Director who is a Director/Officer shall be automatically removed as an officer of the Corporation upon his or her removal as a Director.

#### Section 5: Place of meeting.

The Board of Directors may hold their meetings and have one or more offices, and keep the books of the Corporation, either within or outside the State of California, at such place or places as they may from time to time determine by resolution or by written consent of all the Directors. The Board of Directors may hold their meetings in person, by conference telephone, by E-mail over the Internet or other similar electronic media, or by any combination of the foregoing.

#### Section 6: Regular meetings.

Regular meetings of the Board of Directors may be held at such time and place as shall from time to time be determined by resolution of the Board, provided that written notice of each meeting of the Board of Directors shall be mailed, postage prepaid by the Secretary or person appointed by the President or sent by E-mail over the Internet or similar electronic media by the Secretary or person appointed by the President, to each Director and Director/Officer at his or her post office address or E-mail address, as it appears upon the books of the Corporation, at least ten (10) days before the meeting. The annual meeting of the Board of Directors shall be held within ten days prior to or following the annual meeting of members. Any business may be transacted at any regular meeting of the Board.

#### Section 7: Special meetings.

Special meetings of the Board of Directors shall be held whenever called by any member of the Board of Directors. The Secretary or person appointed by the President shall give notice of each special meeting of the Board of Directors, by mailing the same at least three (3) days prior to the meeting or by E-mailing over the Internet or similar electronic media, the same at least two (2) days before the meeting, to each Director; but such notice may be waived by any Director. Unless otherwise indicated in the notice thereof, any and all business may be transacted at any special meetings. At any meeting at which every Director shall be in Attendance, even though without notice, any business may be transacted and any Director may in writing or by E-mail over the Internet or similar electronic media, waive notice of the time, place and objectives of any special meeting.

####  Section 8: Quorum.

The presence in person, by conference call, by E-mail over the Internet or similar electronic media, or by proxy appointed by an instrument in writing subscribed by such Director or his or her duly authorized attorney, bearing a date not more than three (3) months prior to said meeting, unless said instrument provides for a longer period (such proxy shall be dated, but need not be sealed, witnessed or acknowledged) (each of which shall constitute “Attendance” for all purposes of these bylaws) of a majority of the whole number of Directors shall constitute a quorum for the transaction of business at all meetings of the Board of Directors. If at any meeting less than a quorum shall be in Attendance, a majority of those in Attendance may adjourn the meeting from time to time. The act of a majority of the Directors in Attendance at any meeting at which there is a quorum shall be the act of the Board of Directors, except as may be otherwise specifically provided by law, by the Certificate of Incorporation or by these bylaws.

#### Section 9: Required vote.

An affirmative vote of a majority of those in Attendance shall be necessary for the passage of any resolution.

#### Section 10: Compensation of directors.

Directors, including Director/Officers, shall not receive any stated salary for their services as such, but, at the discretion and unanimous approval by the Executive Committee, a fixed sum may be allowed for Attendance at each regular or special meeting of the Board and such compensation shall be payable whether or not a meeting is adjourned because of the absence of a quorum. This sum may be disbursed to all Directors, including Director/Officers, or to individual Directors or Director/Officers with extenuating circumstances regarding lack of institutional reimbursement of costs for Attendance at each regular or special meeting. If the sum to any or all of the Directors and Director/Officers exceeds $5000, it must be approved by majority vote of the Board. Nothing herein contained shall be construed to preclude any Director or Director/Officer from serving the Corporation in any other capacity, and receiving compensation therefore.

#### Section 11: Standing committees.

The Board of Directors shall elect a septe Standing Committee for the conference coordination for the annual ISMB meeting which is responsible for oversight of all ISCB conference activities sponsored by and affiliated with the Corporation. Another Standing Committee shall be elected to assume responsibility for publications of the society. A Task Force shall be selected for the election of officers and for the setting of standards for voting at meetings held on the Internet or by other electronic media and shall consist of such Directors and members as determined by majority vote of the Directors. The Standing Committees shall be selected by the Board of Directors at any meeting of the Board of Directors. Any and all Task Forces may be selected by the Board of Directors at any duly constituted meeting of the Directors or by the Committee Chair(s) at any committee meeting.

#### Section 12: Other committees.

The Board of Directors may, by resolution passed by a majority of the whole Board, designate one or more other committees, each committee to consist of one or more of the Directors of the Corporation, which, to the extent provided in the resolution, shall have and may exercise the powers of the Board of Directors, and may authorize the seal of the Corporation to be affixed to all papers which may require it. Such committee or committees shall have such names as may be determined from time to time by resolution adopted by the Board of Directors.

#### Section 13: Task forces.

The Board of Directors and committee chairs may, by resolution passed by a majority of the whole Board or at the direction of the committee chairs, designate one or more Task Forces, each Task Force to consist of one or more of the Directors of the Corporation or one or more of the committee members for which the Task Force shall be formed. Task Forces shall be formed for the purposes of task-specific time-limited work in order to make recommendations to the Board of Directors or committee chairs. Task Forces shall not have nor exercise the powers of the Board of Directors, nor authorize the seal of the Corporation to be affixed to any papers which may require it. Such Task Forces shall have such names as may be determined from time to time by resolution adopted by the Board of Directors or at the direction of the committee chairs for which they have formed.

### Article IV: Officers

#### Section 1: Election, tenure and compensation.

The officers of the Corporation shall be a President-elect, a President, a Vice President, a Secretary, and a Treasurer (the “Nominated Officers”), and also such other officers including a Chairman of the Board and/or one or more Vice Presidents and/or one or more assistants to the foregoing officers as the Board of Directors from time to time may appoint for the proper conduct of the business of the Corporation, including but not limited to an Executive Officer to assist in day to day business matters and a Vice Chair of Conferences to assist in managing the annual conference (collectively the “Appointed Officers”). The Nominated Officers shall be nominated by the members and Directors according to the procedures set forth in the Procedure For Nomination. The Nominated Officers shall serve for two years as officers. Any two or more of the above offices, except those of President and Secretary, may be held by the same person, but no officer shall execute, acknowledge or verify any instrument in more than one capacity if such instrument is required by law or by these bylaws to be executed, acknowledged or verified by any two or more officers. If compensation or salary is paid to officers or Director/Officers of the Corporation it shall be fixed by resolutions adopted by the Board of Directors.

In the event that any office other than an office required by law, shall not be filled by the members, or, once filled, subsequently becomes vacant, then such office and all references thereto in these bylaws shall be deemed inoperative unless and until such office is filled in accordance with the provisions of these bylaws.

Except where otherwise expressly provided in a contract duly authorized by the Board of Directors, all officers of the Corporation shall be subject to removal at any time by the affirmative vote of a majority of the Board of Directors or the whole membership at a special meeting of the Board of Directors or the members respectively, duly called according to the rules set forth in these bylaws. All agents and employees of the Corporation shall be subject to removal at any time by the affirmative vote of a majority of the whole Board of Directors, and any agents and employees, other than those elected by the membership, shall hold office at the discretion of the Board of Directors or of the officers appointing them.

#### Section 2: Powers and duties of the chairman of the board.

The Chairman of the Board shall preside at all meetings of the Board of Directors unless the Board of Directors shall by a majority vote of a quorum thereof elect a chairman other than the Chairman of the Board to preside at meetings of the Board of Directors. The Chairman may sign and execute all authorized bonds, contracts or other obligations in the name of the Corporation; and he or she shall be ex-officio a member of all standing committees.

#### Section 3: Powers and duties of the president.

The President shall be the chief executive officer of the Corporation and shall have general charge and control of all its business affairs and properties. He or she shall preside at all meetings of the members.

The President may sign and execute all authorized bonds, contracts or other obligations in the name of the Corporation. The President shall have signature power and the authority to assign signature power to the Executive Officer and/or Vice Chair of Conferences to sign checks in amounts up to $5,000. All checks for amounts over $5,000 for costs not associated with contracts and expenses previously approved by the Board of Directors shall require approval of the Board of Directors and the signature of two officers or one officer and one Appointed Officer. The President shall have the general powers and duties of supervision and management usually vested in the office of president of a corporation. The President shall be ex-officio a member of all the Standing committees. He shall do and perform such other duties as may, from time to time, be assigned to him by the Board of Directors.

In the event that the membership does not take affirmative action to fill the office of Chairman of the Board, the President shall assume and perform all powers and duties given to the Chairman of the Board by these bylaws.

#### Section 4: Powers and duties of the president-elect.

The President-elect may sign and execute all authorized bonds, contracts, or other obligations in the name of the Corporation. The President-elect shall have such other powers and shall perform such other duties as may be assigned to the President-elect by the Board of Directors or by the President. In case of the absence or disability of the President, the duties of that office shall be performed by the President-elect, and the taking of any action by the President-elect in place of the President shall be conclusive evidence of the absence or disability of the President.

#### Section 5: Powers and duties of the vice president.

The Board of Directors may appoint more than one Vice President. Any Vice President (unless otherwise provided by resolution of the Board of Directors) may sign and execute all authorized bonds, contracts, or other obligations in the name of the Corporation. Each Vice President shall have such other powers and shall perform such other duties as may be assigned to the Vice President by the Board of Directors or by the President. In case of the absence or disability of the President and President-elect, the duties of the office of President shall be performed by any Vice President, and the taking of any action by any such Vice President in place of the President shall be conclusive evidence of the absence or disability of the President.

#### Section 6: Secretary.

The Secretary shall handle all voting matters, whether at actual meetings, telephonic meetings or meetings held on the Internet or other electronic media; he or she shall give, or cause to be given, notice of all meetings of members and Directors and all other notices required by law or by these bylaws, and in case of his or her absence or refusal or neglect to do so, any such notice may be given by any person thereunto directed by the President, or by the Directors or members upon whose written request the meeting is called as provided in these bylaws. The Secretary shall record all the proceedings of the meetings of the members and of the Directors in books provided for that purpose, and he or she shall perform such other duties as may be assigned to him or her by the Directors or the President. He or she shall have custody of the seal of the Corporation and shall affix the same to all instruments requiring it, when authorized by the Board of Directors or the President, and attest the same. In general, the Secretary shall perform all the duties generally incident to the office of Secretary, subject to the control of the Board of Directors and the President.

#### Section 7: Treasurer.

The Treasurer shall oversee the Executive Officer's and/or Vice Chair of Conferences' custody of all the funds and securities of the Corporation, and he or she shall oversee the Executive Officer's and/or Vice Chair of Conferences' full and accurate account of receipts and disbursements in books belonging to the Corporation. The Treasurer shall oversee the Appointed Officers' deposit of all moneys and other valuables in the name and to the credit of the Corporation in such depository or depositories as may be designated by the Board of Directors. He or she shall have the power to sign checks under his or her signature in amounts up to $5,000. All checks for amounts over $5,000 shall require the signature of two officers or one officer and one Appointed Officer.

The Treasurer shall oversee the Appointed Officers' disbursement of the funds of the Corporation as may be ordered by the Board of Directors and may require the Appointed Officers to make proper vouchers for such disbursements. The Treasurer shall render to the President and the Board of Directors, whenever either of them so requests, an account of all of the Appointed Officers' transactions and of the financial condition of the Corporation.

The Treasurer shall give the Corporation a bond, if required by the Board of Directors, in a sum, and with one or more sureties, satisfactory to the Board or Directors, for the faithful performance of the duties of his or her office and for the restoration to the Corporation in case of his or her death, resignation, retirement or removal from office of all books, papers, vouchers, moneys, and other properties of whatever kind in his or her possession or under his or her control belonging to the Corporation.

The Treasurer shall perform all the duties generally incident to the office of the Treasurer, subject to the control of the Board of Directors and the President.

#### Section 8: Assistant secretary.

The Board of Directors may appoint an Assistant Secretary or more than one Assistant Secretary. Each Assistant Secretary shall (except as otherwise provided by resolution of the Board of Directors) have power to perform all duties of the Secretary in the absence or disability of the Secretary and shall have such other powers and shall perform such other duties as may be assigned to him by the Board of Directors or the President. In case of the absence or disability of the Secretary, the duties of the office shall be performed by any such Assistant Secretary, and the taking of any action by any such Assistant Secretary in place of the Secretary shall be conclusive evidence of the absence or disability of the Secretary.

#### Section 9: Assistant treasurer.

The Board of Directors may appoint an Assistant Treasurer or more than one Assistant Treasurer. Each Assistant Treasurer shall (except as otherwise provided by resolution of the Board of Directors) have power to perform all duties of the Treasurer in the absence or disability of the Treasurer and shall have such other powers and shall perform such other duties as may be assigned to him by the Board of Directors or the President. In case of the absence or disability of the Treasurer, the duties of the office shall be performed by any Assistant Treasurer, and the taking of any action by any such Assistant Treasurer in place of the Treasurer shall be conclusive evidence of the absence or disability of the Treasurer.

### Article V: Corporate Seal

#### Section 1: Seal.

In the event that the President shall direct the Secretary to obtain a corporate seal, the corporate seal shall be circular in form and shall have inscribed thereon the name of the Corporation, the year of its organization and the word “Delaware”. Duplicate copies of the corporate seal may be provided for use in the different offices of the Corporation but each copy thereof shall be in the custody of the Secretary of the Corporation or of an Assistant Secretary of the Corporation nominated by the Secretary.

### Article VI: Bank Accounts and Loans

#### Section 1: Bank accounts.

Such officers or agents of the Corporation as from time to time shall be designated by the Board of Directors shall have authority to deposit any funds of the Corporation in such banks or trust companies as shall from time to time be designated by the Board of Directors and such officers or agents as from time to time shall be authorized by the Board of Directors to withdraw any or all of the funds of the Corporation so deposited in any such bank or trust company, upon checks, drafts or other instruments or orders for the payment of money, drawn against the account or in the name or behalf of this Corporation, and made or signed by such officers or agents; and each bank or trust company with which funds of the Corporation are so deposited is authorized to accept, honor, cash and pay, without limit as to amount, all checks, drafts or other instruments or orders for the payment of money, when drawn, made or signed by officers or agents so designated by the Board of Directors until written notice of the revocation of the authority of such officers or agents by the Board of Directors shall have been received by such bank or trust company. There shall from time to time be certified to the banks or trust companies in which funds of the Corporation are deposited, the signature of the officers or agents of the Corporation so authorized to draw against the same. In the event that the Board of Directors shall fail to designate the persons by whom checks, drafts and other instruments or orders for the payment of money shall be signed, as hereinabove provided in this Section, all of such checks, drafts and other instruments or orders for the payment of money shall be signed by the President or a Vice President and countersigned by the Secretary or Treasurer or an Assistant Secretary or an Assistant Treasurer or Appointed Officer of the Corporation.

#### Section 2: Loans.

Such officers or agents of this Corporation as from time to time shall be designated by the Board of Directors shall have authority to effect loans, advances or other forms of credit at any time or times for the Corporation from such banks, trust companies, institutions, corporations, firms or persons as the Board or Directors shall from time to time designate, and as security for the repayment of such loans, advances, or other forms of credit to assign, transfer, endorse and deliver, either originally or in addition or substitution, any or all stocks, bonds, rights and interests of any kind in or to stocks or bonds, certificates of such rights or interests, deposits, accounts, documents covering merchandise, deposits and accounts receivable and other commercial paper and evidences of debt at any time held by the Corporation; and for such loans, advances or other forms of credit to make, execute and deliver one or more notes, acceptances or written obligations of the Corporation on such terms, and with such provisions as to the security or sale or disposition thereof as such officers or agents shall deem proper; and also to sell to, or discount or rediscount with, such banks, trust companies, institutions, corporations, firms or persons any and all commercial paper, bills receivable, acceptances and other instruments and evidences of debt at any time held by the Corporation, and to that end to endorse, transfer and deliver the same. There shall from time to time be certified to each bank, trust company, institution, corporation, firm or person so designated the signatures of the officers or agents so authorized; and each such bank, trust company, institution, corporation, firm or person is authorized to reply upon such certification until written notice of the revocation by the Board of Directors of the authority of such officers or agents shall be delivered to such bank, trust company, institution, corporation, firm or person.

### Article VII: Reimbursements

Any payments made to an officer or other employee of the Corporation, such as salary, commission, interest or rent, or entertainment expense incurred by him or her, which shall be disallowed in whole or in part as a deductible expense by the Internal Revenue Service, shall be reimbursed by such officer or other employee of the Corporation to the full extent of such disallowance. It shall be the duty of the Directors, as a Board, to enforce payment of each such amount disallowed. In lieu of payment by the officer or other employee, subject to the determination of the Board of Directors, proportionate amounts may be withheld from his or her future compensation payments until the amount owed to the Corporation has been recovered.

### Article VIII: Miscellaneous Provisions

#### Section 1: Fiscal year.

The fiscal year of the Corporation shall end on the last day of December.

#### Section 2: Notices.

Whenever, under the provisions of these bylaws, notice is required to be given to any Director, officer or member it shall not be construed to mean personal notice, but such notice shall be given in writing, by E-mail over the Internet or similar electronic media, by mail, by depositing the same in a post office or letter box, in a postpaid sealed wrapper, addressed to each member, officer or Director at such address as appears on the books of the Corporation, or in default of any other address, to such Director, officer or member at the general post office in the town of La Jolla, California, and such notice shall be deemed to be given at the time the same shall be thus mailed. Any member, Director or officer may waive any notice required to be given under these bylaws.

#### Section 3: Waiver, consent.

Any notice required to be given under these bylaws or otherwise may be waived by the Director, officer or member to whom such notice is required to be given and the Attendance of any person at a meeting shall constitute waiver of notice thereof as to such person. Any action which may be taken at a meeting of the Directors, officers or members may be taken without a meeting if a consent in writing, setting forth the action so taken, shall be signed by all of the Directors, officers or members entitled to vote with respect to the subject matter thereof. Such consent shall have the same force and effect as a unanimous vote of the Directors, officers or members, as the case may be.

### Article IX: Amendments

#### Section 1. Amendment of bylaws.

Any member can propose an amendment of the bylaws by submitting the change to the President. If a majority of the Board of Directors adopts the amendment it shall be adopted.

### Article X: Indemnification

#### Section 1: Indemnification of directors and officers.

The Corporation shall indemnify and advance expenses to a Director, officer or Appointed Officer of the Corporation in connection with a proceeding to the fullest extent permitted by and in accordance with the General Corporation Law of the State of Delaware.

#### Section 2: Indemnification of employees and agents.

With respect to an employee or agent, other than a Director, officer or Appointed Officer of the Corporation, the Corporation may, as determined by the Board of Directors of the Corporation, indemnify and advance expenses to such employee or agent in connection with a proceeding to the extent permitted by and in accordance with the General Corporation Law of the State of Delaware.

September 16, 2003: Bylaws amended by majority vote of the Board of Directors  

